# Supplementary material for: Experimental evaluation of the importance of colonization history in early-life gut microbiota assembly
Source: eLife. 2018 Sep 18;7:e36521. doi: 10.7554/eLife.36521 (PMC6143339; doi:10.7554/eLife.36521)
Supplement: Supplementary file 1. — Linear mixed model analysis was done to determine the effect of colonization order on community assembly (treatments A/B, AB/AB, B/A; values for A and B (both donor and parent mice) are given as a reference). Results are presented as mean ±standard deviation. [file elife-36521-supp1.docx]

**Supplementary File 1 _** Abundance (% of total sequences) of bacterial types significantly impacted by colonization order in WT mice. Linear mixed model analysis was done to determine the effect of colonization order on community assembly (treatments A/B, AB/AB, B/A; Values for A and B (both donor and parent mice) are given as a reference). Results are presented as mean ± standard deviation.

|  | **A** | **B** | **A/B** | **AB/AB** | **B/A** | **P-value (FDR corrected)** | **Type of priority  effect** | **Taxonomy** |
| --- | --- | --- | --- | --- | --- | --- | --- | --- |
| **Firmicutes** |  |  |  |  |  |  |  |  |
| Type_0857 | 0.00 ± 0.00 | 1.61 ± 1.41 | 0.00 ± 0.00 **^a^** | 0.83 ± 0.68 **^b^** | 1.56 ± 1.62 **^b^** | 8.77 x 10^-04^ | Inhibitory | *Lactobacillus murinus* |
| Type_4146 | 0.00 ± 0.00 | 0.67 ± 0.58 | 0.00 ± 0.00 **^a^** | 0.35 ± 0.28 **^b^** | 0.65 ± 0.66 **^b^** | 8.77 x 10^-04^ | Inhibitory | *Lactobacillus murinus* |
| Type_5899 | 0.00 ± 0.00 | 0.11 ± 0.10 | 0.00 ± 0.01 **^a^** | 0.18 ± 0.13 **^b^** | 0.12 ± 0.07 **^b^** | 9.44 x 10^-03^ | Inhibitory | Ruminococcaceae |
| Type_5446 | 0.00 ± 0.00 | 0.07 ± 0.04 | 0.01 ± 0.01 **^a^** | 0.07 ± 0.07 **^b^** | 0.10 ± 0.05 **^b^** | 9.44 x 10^-03^ | Inhibitory | Firmicutes |
| Type_4364 | 0.00 ± 0.00 | 0.05 ± 0.05 | 0.00 ± 0.00 **^a^** | 0.05 ± 0.20 **^a^** | 0.40 ± 0.26 **^b^** | 1.31 x 10^-02^ | Inhibitory | Ruminococcaceae |
| Type_2038 | 0.00 ± 0.00 | 1.84 ± 1.56 | 0.05 ± 0.08 **^a^** | 0.57 ± 0.52 **^b^** | 0.55 ± 0.36 **^b^** | 1.36 x 10^-02^ | Inhibitory | Lachnospiraceae |
| Type_4547 | 0.00 ± 0.00 | 0.08 ± 0.06 | 0.00 ± 0.00 **^a^** | 0.01 ± 0.01 **^b^** | 0.01 ± 0.01 **^b^** | 1.36 x 10^-02^ | Inhibitory | Lachnospiraceae |
| Type_5505 | 0.00 ± 0.00 | 0.21 ± 0.15 | 0.00 ± 0.00 **^a^** | 0.03 ± 0.04 **^ab^** | 0.09 ± 0.11 **^b^** | 4.79 x 10^-02^ | Inhibitory | Ruminococcaceae |
| Type_5397 | 0.00 ± 0.00 | 0.08 ± 0.07 | 0.00 ± 0.00 **^a^** | 0.03 ± 0.03 **^b^** | 0.03 ± 0.02 **^b^** | 1.67 x 10^-02^ | Inhibitory | Ruminococcaceae |
| Type_4996 | 0.52 ± 0.27 | 0.00 ± 0.00 | 0.97 ± 0.80 **^a^** | 0.09 ± 0.09 **^b^** | 0.03 ± 0.06 **^c^** | 1.66 x 10^-02^ | Inhibitory | Lachnospiraceae |
| Type_1231 | 0.17 ± 0.10 | 0.00 ± 0.00 | 0.16 ± 0.11 **^a^** | 0.05 ± 0.04 **^b^** | 0.01 ± 0.01 **^c^** | 1.66 x 10^-02^ | Inhibitory | Firmicutes |
| Type_5450 | 0.19 ± 0.09 | 0.00 ± 0.00 | 0.15 ± 0.13 **^a^** | 0.05 ± 0.04 **^b^** | 0.01 ± 0.01 **^c^** | 2.20 x 10^-02^ | Inhibitory | Firmicutes |
| Type_2399 | 0.05 ± 0.06 | 0.00 ± 0.00 | 0.19 ± 0.09 **^a^** | 0.20 ± 0.19 **^a^** | 0.02 ± 0.02 **^b^** | 2.20 x 10^-02^ | Inhibitory | Ruminococcaceae |
| Type_4500 | 0.09 ± 0.07 | 0.00 ± 0.00 | 0.02 ± 0.02 **^a^** | 0.11 ± 0.14 **^ab^** | 0.13 ± 0.13 **^b^** | 2.85 x 10^-02^ | Facilitative | *Blautia* |
| Type_5906 | 0.12 ± 0.16 | 0.00 ± 0.00 | 0.13 ± 0.09 **^a^** | 0.10 ± 0.10 **^ab^** | 0.02 ± 0.02 **^b^** | 3.05 x 10^-02^ | Inhibitory | *Oscillibacter* |
| Type_4501 | 0.04 ± 0.03 | 0.00 ± 0.00 | 0.01 ± 0.01 **^a^** | 0.06 ± 0.07 **^ab^** | 0.06 ± 0.06 **^b^** | 4.66 x 10^-02^ | Facilitative | Lachnospiraceae |
| **Bacteroidetes** |  |  |  |  |  |  |  |  |
| Type_5243 | 0.00 ± 0.00 | 1.00 ± 1.10 | 0.00 ± 0.00 **^a^** | 0.14 ± 0.14 **^b^** | 0.25 ± 0.24 **^b^** | 4.94 x 10^-02^ | Inhibitory | Bacteroidetes |
| Type_2870 | 0.00 ± 0.00 | 0.25 ± 0.23 | 0.00 ± 0.00 **^a^** | 0.32 ± 0.29 **^b^** | 0.52 ± 0.27 **^b^** | 7.85 x 10^-04^ | Inhibitory | *Butyricicoccus* |
| Type_4299 | 0.02 ± 0.02 | 3.41 ± 0.89 | 0.00 ± 0.00 **^a^** | 0.25 ± 0.49 **^b^** | 1.25 ± 1.26 **^b^** | 4.79 x 10^-02^ | Inhibitory | Porphyromonadaceae |
| **Actinobacteria** |  |  |  |  |  |  |  |  |
| Type_6280 | 0.00 ± 0.00 | 0.02 ± 0.02 | 0.00 ± 0.00 **^a^** | 0.03 ± 0.02 **^b^** | 0.04 ± 0.03 **^b^** | 8.77 x 10^-04^ | Inhibitory | Coriobacteriaceae |
