# Supplementary material for: Experimental evaluation of the importance of colonization history in early-life gut microbiota assembly
Source: eLife. 2018 Sep 18;7:e36521. doi: 10.7554/eLife.36521 (PMC6143339; doi:10.7554/eLife.36521)
Supplement: Supplementary file 2. — Results are presented as mean ±standard deviation). [file elife-36521-supp2.docx]

**Supplementary File 2** _ Abundance (% of total sequences) of bacterial types significantly impacted by inoculation time of four specific colonizing strains in WT mice, assessed through Random Forest analysis (Random Forest coefficient >2 was considered significant). Results are presented as mean ± standard deviation.

| **Node** | **Group 1 (Day 5 inoc)** | **Group 2 (Day 14 inoc)** | **Group 3 (Day 36 inoc)** | **Mean importance  Random Forest** | **Taxonomic classification** |
| --- | --- | --- | --- | --- | --- |
| **Firmicutes** |  |  |  |  |  |
| Type_0920 | 0.06 ± 0.04 | 0.09 ± 0.11 | 0.37 ± 0.33 | 4.581 | Lachnospiraceae |
| Type_3101 | 0.21 ± 0.04 | 0.10 ± 0.06 | 0.07 ± 0.05 | 4.479 | Ruminococcaceae |
| Type_3049 | 0.39 ± 0.22 | 0.12 ± 0.10 | 0.16 ± 0.10 | 4.169 | *Lactobacillus panis* |
| Type_3544 | 0.21 ± 0.16 | 0.09 ± 0.07 | 0.21 ± 0.10 | 3.837 | Lachnospiraceae |
| Type_0060 | 0.27 ± 0.11 | 0.49 ± 0.31 | 0.29 ± 0.09 | 3.703 | *Clostridium lactatifermentans* |
| Type_3875 | 0.05 ± 0.05 | 0.03 ± 0.07 | 0.07 ± 0.03 | 3.583 | *Clostridium scindens* |
| Type_2592 | 0.09 ± 0.04 | 0.05 ± 0.05 | 0.00 ± 0.01 | 3.265 | Lachnospiraceae |
| Type_0091 | 0.03 ± 0.02 | 0.10 ± 0.08 | 0.08 ± 0.04 | 2.925 | Clostridiales |
| Type_2375 | 0.25 ± 0.51 | 0.02 ± 0.02 | 0.06 ± 0.05 | 2.834 | Lachnospiraceae |
| Type_0913 | 0.25 ± 0.33 | 0.29 ± 0.48 | 0.01 ± 0.01 | 2.645 | Lachnospiraceae |
| Type_3693 | 0.07 ± 0.06 | 0.03 ± 0.05 | 0.14 ± 0.10 | 2.627 | Lactobacillaceae |
| Type_2530 | 0.25 ± 0.10 | 0.14 ± 0.07 | 0.11 ± 0.09 | 2.597 | Lachnospiraceae |
| Type_1712 | 0.00 ± 0.00 | 0.05 ± 0.08 | 0.08 ± 0.07 | 2.577 | Firmicutes |
| Type_4071 | 0.09 ± 0.10 | 0.03 ± 0.03 | 0.12 ± 0.10 | 2.514 | *Lactobacillus panis* |
| Type_3233 | 1.87 ± 0.65 | 1.03 ± 0.50 | 1.52 ± 1.32 | 2.476 | Lachnospiraceae |
| Type_3857 | 0.04 ± 0.01 | 0.12 ± 0.10 | 0.09 ± 0.04 | 2.440 | *Dorea* |
| Type_4155 | 0.09 ± 0.11 | 0.03 ± 0.03 | 0.11 ± 0.10 | 2.396 | *Lactobacillus johnsonii/gasseri* |
| Type_3931 | 0.13 ± 0.23 | 0.01 ± 0.02 | 0.02 ± 0.03 | 2.333 | Lachnospiraceae |
| Type_2180 | 0.23 ± 0.23 | 0.24 ± 0.19 | 0.07 ± 0.08 | 2.190 | Lachnospiraceae |
| Type_0610 | 0.00 ± 0.00 | 0.00 ± 0.00 | 0.15 ± 0.19 | 2.170 | Lachnospiraceae |
| Type_2597 | 0.02 ± 0.03 | 0.04 ± 0.03 | 0.01 ± 0.02 | 2.160 | *Clostridium scindens* |
| Type_2697 | 0.06 ± 0.04 | 0.11 ± 0.12 | 0.07 ± 0.05 | 2.005 | *Oscillibacter* |
| **Bacteroidetes** |  |  |  |  |  |
| Type_4159 | 0.02 ± 0.03 | 0.21 ± 0.12 | 0.15 ± 0.14 | 5.471 | Bacteroidales |
| Type_0110 | 0.01 ± 0.02 | 0.06 ± 0.04 | 0.07 ± 0.04 | 2.681 | *Bacteroides stercoris* |
| **Actinobacteria** |  |  |  |  |  |
| Type_2455 | 0.47 ± 0.21 | 0.84 ± 0.31 | 0.35 ± 0.26 | 4.145 | Coriobacteriaceae |
| **Verrucomicrobia** |  |  |  |  |  |
| Type_0100 | 0.04 ± 0.07 | 0.32 ± 0.52 | 0.38 ± 0.29 | 2.898 | *Akkermansia muciniphila* |
