# Supplementary material for: Experimental evaluation of the importance of colonization history in early-life gut microbiota assembly
Source: eLife. 2018 Sep 18;7:e36521. doi: 10.7554/eLife.36521 (PMC6143339; doi:10.7554/eLife.36521)
Supplement: Supplementary file 3. — Results are presented as mean ±standard deviation. [file elife-36521-supp3.docx]

**Supplementary File 3** _ Abundance (expressed as percent of total sequences) of bacterial types significantly different between WT and *Rag1^-/-^*, for experiments when the effect of colonization order of two donor cecal communities on the final community composition was tested. Results are presented as mean ± standard deviation.

|  | **WT** | ***Rag1-/-*** | ***P-value* (FDR corrected)** | **Taxonomy** |
| --- | --- | --- | --- | --- |
| **Bacteroidetes** |  |  |  |  |
| Type_0083 | 0.03 ± 0.02 | 0.01 ± 0.01 | 0.015 | *Barnesiella* |
| Type_0445 | 3.77 ± 1.99 | 6.97 ± 2.98 | 0.044 | Bacteroidales |
| Type_2464 | 0.01 ± 0.01 | 0.03 ± 0.03 | 0.019 | Porphyromonadaceae |
| Type_4299 | 0.48 ± 0.92 | 0.78 ± 0.51 | 0.044 | Porphyromonadaceae |
| Type_5851 | 0.06 ± 0.04 | 0.01 ± 0.01 | 0.008 | *Barnesiella* |
| Type_5853 | 0.03 ± 0.03 | 0.01 ± 0.01 | 0.016 | *Barnesiella* |
| **Firmicutes** |  |  |  |  |
| Type_3098 | 0.39 ± 0.37 | 0.13 ± 0.09 | 0.010 | Clostridiales |
| Type_5733 | 0.14 ± 0.14 | 0.05 ± 0.03 | 0.016 | Clostridiales |
| Type_5734 | 0.10 ± 0.10 | 0.04 ± 0.03 | 0.025 | Clostridiales |
| Type_5646 | 0.11 ± 0.15 | 0.02 ± 0.03 | 0.047 | Lachnospiraceae |
| Type_5729 | 0.65 ± 0.84 | 0.10 ± 0.12 | 0.008 | Lachnospiraceae |
| Type_5730 | 1.29 ± 1.87 | 0.18 ± 0.19 | 0.008 | Lachnospiraceae |
| Type_5731 | 0.05 ± 0.07 | 0.01 ± 0.02 | 0.008 | Lachnospiraceae |
| Type_6327 | 0.04 ± 0.06 | 0.01 ± 0.01 | 0.040 | Lachnospiraceae |
| Type_2064 | 0.11 ± 0.10 | 0.04 ± 0.04 | 0.016 | Lachnospiraceae |
| Type_2080 | 0.03 ± 0.02 | 0.01 ± 0.01 | 0.008 | Lachnospiraceae |
| Type_2035 | 0.17 ± 0.07 | 0.11 ± 0.05 | 0.012 | Lachnospiraceae |
| Type_2026 | 0.26 ± 0.28 | 0.06 ± 0.05 | 0.025 | Lachnospiraceae |
| Type_5893 | 0.01 ± 0.01 | 0.02 ± 0.01 | 0.021 | Ruminococcaceae |
| Type_3561 | 2.38 ± 2.76 | 0.49 ± 0.86 | 0.010 | *Turicibacter* |
| Type_3562 | 0.34 ± 0.38 | 0.06 ± 0.11 | 0.008 | *Turicibacter* |
