# Supplementary material for: Experimental evaluation of the importance of colonization history in early-life gut microbiota assembly
Source: eLife. 2018 Sep 18;7:e36521. doi: 10.7554/eLife.36521 (PMC6143339; doi:10.7554/eLife.36521)
Supplement: Supplementary file 4. — Statistical analyses were done for treatments A/B, AB/AB, B/A (A and B are given as a reference) using a linear mixed model with genetic background and colinization order as the main effects. Results are presented as mean ±standard deviation. [file elife-36521-supp4.docx]

**Supplementary File 4 _** Abundance (% of total sequences) of bacterial types significantly impacted by colonization order in *Rag1^-/-^* and WT mice. Statistical analyses were done for treatments A/B, AB/AB, B/A (A and B are given as a reference) using a linear mixed model with genetic background and colonization order as the main effects. Results are presented as mean ± standard deviation.

| **Node** | **A** | **B** | **A/B** | **AB/AB** | **B/A** | ***P*-value** | **Type of priority effects** | **Taxonomy** |
| --- | --- | --- | --- | --- | --- | --- | --- | --- |
|  |  |  |  |  |  | **(FDR corrected)** |  |  |
| **Actinobacteria** |  |  |  |  |  |  |  |  |
| Type_6280 | 0.00 ± 0.00 | 0.02 ± 0.01 | 0.00 ± 0.00**^a^** | 0.03 ± 0.02**^b^** | 0.04 ± 0.03**^b^** | 1.50E-05 | Inhibitory | Coriobacteriaceae |
| **Bacteroidetes** |  |  |  |  |  |  |  |  |
| Type_2472 | 0.64 ± 0.28 | 0.00 ± 0.00 | 0.75 ± 0.37**^a^** | 0.57 ± 0.33**^a^** | 0.35 ± 0.29**^b^** | 4.90E-02 | Inhibitory | *Rikenella* |
| Type_4297 | 0.61 ± 0.29 | 0.01 ± 0.01 | 1.50 ± 0.75**^a^** | 0.92 ± 0.86**^a^** | 0.13 ± 0.34**^b^** | 8.90E-03 | Inhibitory | Porphyromonadaceae |
| Type_4299 | 0.03 ± 0.03 | 3.49 ± 1.43 | 0.20 ± 0.34**^a^** | 0.50 ± 0.60**^b^** | 1.16 ± 0.90**^b^** | 1.60E-02 | Inhibitory | Porphyromonadaceae |
| Type_5242 | 0.00 ± 0.00 | 0.43 ± 0.47 | 0.00 ± 0.01**^a^** | 0.06 ± 0.07**^b^** | 0.08 ± 0.09**^b^** | 8.50E-03 | Inhibitory | Bacteroidetes |
| Type_5243 | 0.00 ± 0.00 | 0.89 ± 0.97 | 0.01 ± 0.02**^a^** | 0.13 ± 0.12**^b^** | 0.17 ± 0.19**^b^** | 7.10E-04 | Inhibitory | Bacteroidetes |
| Type_5289 | 0.10 ± 0.08 | 0.00 ± 0.00 | 0.17 ± 0.15**^a^** | 0.13 ± 0.12**^a^** | 0.06 ± 0.27**^b^** | 5.10E-03 | Inhibitory | *Alistipes* |
| Type_5291 | 1.13 ± 0.69 | 1.15 ± 0.79 | 2.76 ± 1.28**^a^** | 2.39 ± 0.85**^a^** | 1.18 ± 0.67**^b^** | 8.00E-03 | NA | *Alistipes* |
| **Candidatus** |  |  |  |  |  |  |  |  |
| **Saccharibacteria** |  |  |  |  |  |  |  |  |
| Type_3332 | 0.05 ± 0.03 | 0.00 ± 0.00 | 0.04 ± 0.02**^a^** | 0.02 ± 0.02**^b^** | 0.03 ± 0.02**^b^** | 4.30E-02 | Inhibitory | *Saccharibacteria* |
| **Firmicutes** |  |  |  |  |  |  |  |  |
| Type_0575 | 0.05 ± 0.07 | 0.00 ± 0.00 | 0.05 ± 0.05**^a^** | 0.01 ± 0.01**^b^** | 0.01 ± 0.01**^b^** | 2.80E-03 | Inhibitory | Ruminococcaceae |
| Type_0857 | 0.00 ± 0.01 | 1.55 ± 1.22 | 0.00 ± 0.00**^a^** | 1.07 ± 1.26**^b^** | 1.29 ± 1.25**^b^** | 9.90E-06 | Inhibitory | *Lactobacillus* |
| Type_1231 | 0.19 ± 0.14 | 0.00 ± 0.00 | 0.14 ± 0.10**^a^** | 0.05 ± 0.04**^b^** | 0.01 ± 0.01**^c^** | 4.50E-04 | Inhibitory | Firmicutes |
| Type_1817 | 1.15 ± 0.76 | 0.94 ± 0.38 | 0.92 ± 0.71**^a^** | 2.88 ± 1.60**^b^** | 1.44 ± 0.98**^a^** | 7.40E-03 | NA | Lachnospiraceae |
| Type_1831 | 0.02 ± 0.01 | 0.02 ± 0.01 | 0.02 ± 0.01**^a^** | 0.05 ± 0.03**^b^** | 0.02 ± 0.02**^a^** | 1.30E-02 | NA | Lachnospiraceae |
| Type_2003 | 0.00 ± 0.00 | 0.20 ± 0.10 | 0.01 ± 0.02**^a^** | 0.03 ± 0.03**^b^** | 0.05 ± 0.03**^b^** | 6.90E-03 | Inhibitory | Lachnospiraceae |
| Type_2038 | 0.00 ± 0.00 | 2.01 ± 1.38 | 0.04 ± 0.08**^a^** | 0.40 ± 0.43**^b^** | 0.86 ± 0.66**^b^** | 2.10E-03 | Inhibitory | Lachnospiraceae |
| Type_2059 | 0.08 ± 0.04 | 0.00 ± 0.00 | 0.02 ± 0.02**^a^** | 0.00 ± 0.01**^b^** | 0.00 ± 0.00**^b^** | 1.00E-03 | Inhibitory | Lachnospiraceae |
| Type_2233 | 1.19 ± 0.37 | 0.00 ± 0.00 | 0.82 ± 0.34**^a^** | 0.96 ± 0.30**^a^** | 0.58 ± 0.24**^b^** | 1.30E-02 | Inhibitory | Clostridiales |
| Type_2252 | 0.00 ± 0.00 | 0.20 ± 0.11 | 0.01 ± 0.01**^a^** | 0.02 ± 0.01**^b^** | 0.01 ± 0.01**^a^** | 2.90E-03 | NA | Clostridiales |
| **Node** | **A** | **B** | **A/B** | **AB/AB** | **B/A** | ***P*-value(FDR corrected)** | **Type of priority effects** | **Taxonomy** |
| Type_2399 | 0.03 ± 0.05 | 0.00 ± 0.00 | 0.24 ± 0.14**^a^** | 0.21 ± 0.19**^a^** | 0.03 ± 0.04**^b^** | 9.40E-04 | Inhibitory | Ruminococcaceae |
| Type_2870 | 0.00 ± 0.00 | 0.21 ± 0.21 | 0.00 ± 0.00**^a^** | 0.24 ± 0.27**^b^** | 0.46 ± 0.32**^b^** | 9.40E-04 | Inhibitory | *Butyricicoccus* |
| Type_3269 | 0.05 ± 0.04 | 0.00 ± 0.00 | 0.02 ± 0.04**^a^** | 0.14 ± 0.22**^b^** | 0.09 ± 0.05**^b^** | 4.30E-02 | Facilitative | *Clostridium* XI |
| Type_4146 | 0.00 ± 0.00 | 0.63 ± 0.50 | 0.00 ± 0.00**^a^** | 0.42 ± 0.47**^b^** | 0.54 ± 0.51**^c^** | 1.30E-05 | Inhibitory | Lactobacillaceae |
| Type_4364 | 0.00 ± 0.00 | 0.06 ± 0.06 | 0.00 ± 0.00**^a^** | 0.04 ± 0.16**^a^** | 0.35 ± 0.25**^b^** | 1.50E-07 | Inhibitory | Ruminococcaceae |
| Type_4481 | 0.00 ± 0.00 | 0.04 ± 0.03 | 0.00 ± 0.00**^a^** | 0.01 ± 0.01**^a^** | 0.02 ± 0.03**^b^** | 3.50E-02 | Inhibitory | Lachnospiraceae |
| Type_4500 | 0.07 ± 0.06 | 0.00 ± 0.00 | 0.01 ± 0.02**^a^** | 0.08 ± 0.11**^b^** | 0.13 ± 0.14**^b^** | 8.90E-03 | Facilitative | *Blautia* |
| Type_4501 | 0.03 ± 0.03 | 0.00 ± 0.00 | 0.01 ± 0.01**^a^** | 0.04 ± 0.06**^b^** | 0.06 ± 0.07**^b^** | 1.20E-02 | Facilitative | Lachnospiraceae |
| Type_4547 | 0.00 ± 0.00 | 0.08 ± 0.07 | 0.00 ± 0.00**^a^** | 0.01 ± 0.01**^a,b^** | 0.01 ± 0.01**^b^** | 3.70E-02 | Inhibitory | Lachnospiraceae |
| Type_4786 | 0.00 ± 0.00 | 0.06 ± 0.04 | 0.00 ± 0.00**^a^** | 0.01 ± 0.01**^b^** | 0.02 ± 0.01**^b^** | 6.30E-03 | Inhibitory | Lachnospiraceae |
| Type_4996 | 0.58 ± 0.24 | 0.00 ± 0.00 | 0.80 ± 0.64**^a^** | 0.10 ± 0.16**^b^** | 0.03 ± 0.05**^c^** | 4.40E-05 | Inhibitory | Lachnospiraceae |
| Type_4997 | 0.00 ± 0.01 | 1.12 ± 1.13 | 0.07 ± 0.28**^a^** | 1.49 ± 1.06**^b^** | 1.27 ± 1.41**^b^** | 1.50E-04 | Inhibitory | Lachnospiraceae |
| Type_5149 | 0.02 ± 0.02 | 0.00 ± 0.00 | 0.07 ± 0.05**^a^** | 0.00 ± 0.01**^b^** | 0.01 ± 0.04**^b^** | 2.80E-04 | Inhibitory | Clostridia |
| Type_5397 | 0.00 ± 0.00 | 0.06 ± 0.06 | 0.00 ± 0.01**^a^** | 0.03 ± 0.02**^b^** | 0.02 ± 0.02**^b^** | 5.20E-03 | Inhibitory | Ruminococcaceae |
| Type_5446 | 0.00 ± 0.00 | 0.08 ± 0.04 | 0.00 ± 0.01**^a^** | 0.06 ± 0.07**^b^** | 0.09 ± 0.06**^c^** | 2.10E-04 | Inhibitory | Firmicutes |
| Type_5450 | 0.19 ± 0.09 | 0.00 ± 0.00 | 0.14 ± 0.11**^a^** | 0.06 ± 0.05**^b^** | 0.01 ± 0.01**^c^** | 5.10E-04 | Inhibitory | Firmicutes |
| Type_5462 | 0.00 ± 0.00 | 0.23 ± 0.75 | 0.00 ± 0.00**^a^** | 0.00 ± 0.00**^a^** | 0.00 ± 0.01**^b^** | 1.30E-02 | Inhibitory | Streptococcaceae |
| Type_5775 | 0.18 ± 0.18 | 0.00 ± 0.00 | 0.06 ± 0.13**^a^** | 0.45 ± 0.78**^b^** | 0.26 ± 0.18**^b^** | 4.90E-02 | Facilitative | *Clostridium* XI |
| Type_5899 | 0.00 ± 0.00 | 0.10 ± 0.09 | 0.00 ± 0.01**^a^** | 0.25 ± 0.16**^b^** | 0.16 ± 0.10**^b^** | 3.80E-06 | Inhibitory | Ruminococcaceae |
| Type_5906 | 0.07 ± 0.12 | 0.00 ± 0.00 | 0.16 ± 0.13**^a^** | 0.10 ± 0.09**^a^** | 0.03 ± 0.03**^b^** | 3.20E-02 | Inhibitory | Ruminococcaceae |
| Type_6103 | 0.09 ± 0.05 | 0.00 ± 0.00 | 0.03 ± 0.05**^a^** | 0.02 ± 0.03**^a^** | 0.00 ± 0.01**^b^** | 4.80E-02 | Inhibitory | Lachnospiraceae |
| Type_6301 | 0.00 ± 0.00 | 0.04 ± 0.03 | 0.07 ± 0.04**^a,b^** | 0.10 ± 0.04**^b^** | 0.06 ± 0.04**^a^** | 2.90E-02 | Inhibitory | Ruminococcaceae |
| Type_6326 | 0.19 ± 0.39 | 0.02 ± 0.02 | 0.32 ± 0.31**^a^** | 0.10 ± 0.17**^a^** | 0.10 ± 0.11**^b^** | 5.00E-02 | Inhibitory | Lachnospiraceae |
| **Proteobacteria** |  |  |  |  |  |  |  |  |
| Type_2069 | 0.03 ± 0.09 | 0.00 ± 0.00 | 0.04 ± 0.04**^a^** | 0.00 ± 0.00**^b^** | 0.00 ± 0.00**^b^** | 7.80E-03 | Inhibitory | Enterobacteriaceae |
