# Supplementary material for: Experimental evaluation of the importance of colonization history in early-life gut microbiota assembly
Source: eLife. 2018 Sep 18;7:e36521. doi: 10.7554/eLife.36521 (PMC6143339; doi:10.7554/eLife.36521)
Supplement: Supplementary file 5. — Results are presented as mean ± standard deviation. Taxa in bold were also impacted in WT mice (Supplementary File 2) [file elife-36521-supp5.docx]

**Supplementary File 5 _** Abundance (% of total sequences) of bacterial types significantly impacted by inoculation time of specific colonizing strains in *Rag1^-/-^* mice, assessed through Random Forest analysis (Random Forest coefficient ≥2 was considered significant). Results are presented as mean ± standard deviation. Taxa in bold were also impacted in WT mice (Supplementary File 2).

| **Node** | **Group 1 (Day 5 inoc)** | **Group 2 (Day 14 inoc)** | **Group 3 (Day 36 inoc)** | **Mean importance  Random Forest** | **Taxonomic classification** |
| --- | --- | --- | --- | --- | --- |
| **Firmicutes** |  |  |  |  |  |
| Type_0002 | 0.06 ± 0.03 | 0.01 ± 0.02 | 0.04 ± 0.05 | 2.11 | Ruminococcaceae |
| Type_0042 | 0.14 ± 0.10 | 0.07 ± 0.07 | 0.20 ± 0.08 | 3.29 | Firmicutes |
| Type_0056 | 0.01 ± 0.02 | 0.08 ± 0.10 | 0.24 ± 0.25 | 2.00 | *Enterococcus durans* |
| Type_0057 | 0.07 ± 0.06 | 0.05 ± 0.08 | 0.06 ± 0.07 | 2.06 | Erysipelotrichaceae |
| **Type_0060** | **0.44 ± 0.14** | **0.37 ± 0.30** | **0.15 ± 0.07** | **3.69** | ***Clostridium lactatifermentans*** |
| Type_0063 | 0.02 ± 0.02 | 0.01 ± 0.01 | 0.06 ± 0.06 | 2.34 | *Lactobacillus* sp. |
| Type_0068 | 0.13 ± 0.13 | 0.07 ± 0.10 | 0.13 ± 0.06 | 3.41 | *Eubacterium* sp. |
| Type_0078 | 0.09 ± 0.06 | 0.22 ± 0.08 | 0.17 ± 0.06 | 3.93 | *Parasutterella excrementihominis* |
| Type_0145 | 0.21 ± 0.19 | 0.03 ± 0.05 | 0.01 ± 0.01 | 2.23 | *Clostridium* sp. |
| Type_0516 | 0.04 ± 0.02 | 0.00 ± 0.00 | 0.01 ± 0.01 | 4.22 | Erysipelotrichaceae |
| **Type_0610** | **0.58 ± 0.22** | **0.00 ± 0.00** | **0.00 ± 0.00** | **6.82** | **Lachnospiraceae** |
| **Type_0920** | **0.29 ± 0.11** | **0.23 ± 0.21** | **0.08 ± 0.06** | **3.39** | ***Clostridium* sp.** |
| Type_1078 | 1.46 ± 0.65 | 0.79 ± 0.42 | 0.59 ± 0.27 | 2.00 | Clostridiales |
| Type_1236 | 0.51 ± 0.44 | 0.51 ± 0.80 | 0.46 ± 0.12 | 2.40 | Ruminococcaceae |
| Type_1418 | 0.03 ± 0.02 | 0.06 ± 0.06 | 0.09 ± 0.04 | 2.46 | Lachnospiraceae |
| Type_1465 | 0.06 ± 0.04 | 0.02 ± 0.03 | 0.04 ± 0.02 | 2.00 | Firmicutes |
| Type_1759 | 0.32 ± 0.15 | 0.36 ± 0.23 | 0.12 ± 0.07 | 2.42 | Ruminococcaceae |
| Type_1760 | 0.45 ± 0.15 | 0.75 ± 0.30 | 0.40 ± 0.31 | 2.42 | Ruminococcaceae |
| Type_1909 | 0.11 ± 0.06 | 0.12 ± 0.05 | 0.07 ± 0.07 | 2.24 | *Pseudoflavonifractor* sp. |
| Type_2022 | 0.32 ± 0.29 | 0.07 ± 0.09 | 0.01 ± 0.02 | 2.36 | Lachnospiraceae |
| Type_2277 | 0.25 ± 0.11 | 0.11 ± 0.06 | 0.30 ± 0.17 | 2.63 | Clostridiales |
| Type_2376 | 0.06 ± 0.03 | 0.09 ± 0.21 | 0.70 ± 1.00 | 2.64 | Lachnospiraceae |
| Type_2377 | 8.21 ± 2.30 | 4.48 ± 2.63 | 2.68 ± 1.71 | 3.24 | Lachnospiraceae |
| Type_2383 | 0.18 ± 0.05 | 0.10 ± 0.07 | 0.07 ± 0.09 | 3.42 | Lachnospiraceae |
| **Type_2597** | **0.15 ± 0.10** | **0.00 ± 0.00** | **0.00 ± 0.00** | **6.97** | ***Clostridium scindens*** |
| Type_2600 | 0.25 ± 0.10 | 0.07 ± 0.02 | 0.20 ± 0.17 | 3.89 | Lachnospiraceae |
| Type_2885 | 0.63 ± 0.30 | 0.65 ± 0.29 | 0.37 ± 0.14 | 2.45 | *Enterorhabdus caecimuris* |
| Type_2887 | 5.37 ± 1.69 | 6.99 ± 2.07 | 4.61 ± 1.10 | 2.17 | Bacteroidetes |
| **Type_3101** | **0.24 ± 0.08** | **0.13 ± 0.06** | **0.13 ± 0.09** | **2.49** | **Ruminococcaceae** |
| Type_3244 | 0.04 ± 0.02 | 0.02 ± 0.02 | 0.01 ± 0.01 | 2.41 | Clostridiales |
| Type_3406 | 0.11 ± 0.09 | 0.08 ± 0.10 | 0.18 ± 0.07 | 2.51 | Clostridium sp. |
| Type_3582 | 0.08 ± 0.06 | 0.02 ± 0.03 | 0.01 ± 0.02 | 2.60 | Clostridiales |
| Type_3583 | 0.19 ± 0.10 | 0.05 ± 0.04 | 0.03 ± 0.02 | 4.22 | Clostridiales |
| Type_3900 | 0.34 ± 0.16 | 0.28 ± 0.15 | 0.17 ± 0.06 | 2.32 | Lachnospiraceae |
| Type_4118 | 0.10 ± 0.06 | 0.03 ± 0.03 | 0.02 ± 0.01 | 5.49 | Lachnospiraceae |
| **Bacteroidetes** |  |  |  |  |  |
| **Type_0110** | **0.02 ± 0.03** | **0.14 ± 0.08** | **0.11 ± 0.08** | **4.03** | ***Bacteroides stercoris*** |
| Type_4003 | 0.02 ± 0.01 | 0.05 ± 0.03 | 0.02 ± 0.02 | 2.02 | Bacteroidetes |
| Type_4152 | 0.05 ± 0.03 | 0.13 ± 0.07 | 0.04 ± 0.04 | 2.63 | *Bacteroides vulgatus* |
| Type_4153 | 0.00 ± 0.00 | 0.02 ± 0.03 | 0.05 ± 0.04 | 2.63 | Bacteroidetes |
| Type_1304 | 0.92 ± 0.35 | 1.61 ± 0.51 | 0.89 ± 0.33 | 3.20 | Bacteroidetes |
| Type_1313 | 2.25 ± 1.12 | 4.49 ± 1.31 | 3.27 ± 1.03 | 2.96 | Bacteroidetes |
| Type_1331 | 0.01 ± 0.01 | 0.07 ± 0.04 | 0.03 ± 0.03 | 4.70 | Bacteroidetes |
| Type_2817 | 0.35 ± 0.07 | 0.42 ± 0.16 | 0.21 ± 0.14 | 2.00 | Bacteroidetes |
| **Type_4159** | **0.08 ± 0.06** | **0.01 ± 0.01** | **0.01 ± 0.02** | **3.14** | **Bacteroidetes** |
| **Actinobacteria** |  |  |  |  |  |
| **Type_2455** | **0.15 ± 0.10** | **0.62 ± 0.41** | **0.50 ± 0.17** | **4.37** | **Coriobacteriaceae** |
| **Verrucomicrobia** |  |  |  |  |  |
| **Type_0100** | **0.02 ± 0.03** | **0.53 ± 0.56** | **0.15 ± 0.15** | **2.74** | ***Akkermansia muciniphila*** |
