# Supplementary material for: Experimental evaluation of the importance of colonization history in early-life gut microbiota assembly
Source: eLife. 2018 Sep 18;7:e36521. doi: 10.7554/eLife.36521 (PMC6143339; doi:10.7554/eLife.36521)
Supplement: Supplementary file 6. [file elife-36521-supp6.docx]

**Supplementary File 6.** Primer-pair used for absolute quantification of the specific colonizer strains.

| **Specific colonizer strains** | **Primer sequence (5' - 3')** | | **T_m_ (°C )** | **Product size (nt)** |
| --- | --- | --- | --- | --- |
| *Lactobacillus reuteri*  Lpuph-1 | Forward | GCGTACATGGAGCGGTAAGA | 59.9 | 220 |
|  | Reverse | AGCCGTTCCACCAATAGCAA | 59.96 |  |
|  |  |  |  |  |
| *Clostridium cocleatum*  ATCC 29902 | Forward | TGTTGGGAATGATGGTGATG | 60.18 | 186 |
|  | Reverse | TAACCAAATGCCCTTTCTGC | 60.07 |  |
|  |  |  |  |  |
| *Lactobacillus johnsonii*  DPPM | Forward | GCATTGGCACATGACTTCAA | 60.68 | 109 |
|  | Reverse | CTGACGGATACCAGCCATTA | 58.6 |  |
|  |  |  |  |  |
| *Bacteroides vulgatus*  RJ2H1 | Forward | ATGGGGAAAGACCGGTAATC | 60.72 | 121 |
|  | Reverse | TCCCGAATATTGCAACAAC | 57.76 |  |
